# Mildly Processed Natural Eggshell Membrane Alleviates Joint Pain Associated with Osteoarthritis of the Knee: A Randomized Double-Blind Placebo-Controlled Study

**DOI:** 10.1089/jmf.2020.0034

**Published:** 2021-03-16

**Authors:** Jeroen Lucas Kiers, Johannes Hendrikus Franciscus Bult

**Affiliations:** ^1^ JLK Nutrition, Amersfoort, The Netherlands.; ^2^ Applegg, Amersfoort, The Netherlands.

**Keywords:** cartilage, knee pain, medicinal food, nutrition therapy

## Abstract

Poor joint health is a significant burden to society. Millions of people suffer from some form of joint-related disorder or disease, most often osteoarthritis (OA). It was hypothesized that chicken eggshell membrane (EM) is effective in the regeneration of cartilage and/or immunomodulation (oral tolerance), and as such relieves pain and stiffness in joints commonly affected in arthritis. We tested this hypothesis in a double-blind, placebo-controlled EM intervention study. Of 150 male and female volunteers, 40–75 years of age and diagnosed with knee OA, 75 were randomly assigned to the EM intervention group and 75 to the placebo group. During 12 weeks, subjects received a daily capsule containing either 300 mg of EM or a placebo. The main primary dependent variable consisted of self-reported pain ratings on a Numerical Rating Scale Pain (NRS-P) 6 weeks after study start. As secondary dependent variables served NRS-P scores collected after 12 weeks, and Knee injury and self-reported Osteoarthritis Outcome Scores (Knee injury and Osteoarthritis Outcome Scores [KOOS]). NRS-P scores decreased for both groups at approximately the same rate, but only EM relieved self-reported pain scores obtained with the KOOS questionnaire starting 1 week after initiation of treatment. This effect was significant for two of five KOOS category scores, that is, “Pain” and “Daily Life” functioning, aggregate pain, and functioning scores composed of complaint ratings for a wide variety of daily activities. These scores showed long-lasting improvement, and demonstrated that EM extract successfully reliefs knee OA pain and contributes to daily life functioning.

## Introduction

Arthritis is a disorder characterized by chronic inflammation in one or more joints. It usually results in pain and is often disabling. Of the more than 100 different manifestations of arthritis, osteoarthritis (OA) is most commonly observed. Other forms include rheumatoid arthritis, psoriatic arthritis, and related autoimmune diseases.^1^ Arthritis disregards age, gender, and ethnicity, and it is the leading cause of disability in many Western countries. Although causes of arthritis remain uncertain, and may vary, its symptoms are rather uniform. Common arthritis symptoms include swelling, pain, stiffness, and limited mobility of the joints. Although the disorder is chronic, its symptoms are not. They can be mild, moderate, or severe. For some patients, symptoms may not change for years, whereas for others they may progress and get worse over time. Severe arthritis can result in chronic pain, preventing patients to take part in daily activities that require walking or climbing stairs. Arthritis can cause permanent joint changes, sometimes visible such as knobby finger joints, but often only to be diagnosed by X-ray.

As OA is a degenerative joint disorder, its incidence is also growing with the progressively aging population. The worldwide prevalence of knee OA increased with 26.6% from 1990 to 2010, and it currently affects about 9.6% of men and 18% of women over 60 years of age.^2^ This age dependency is attributed to the decreased capacity to suppress inflammation, age-related sarcopenia, and increased bone turnover.^1^

Yet, the pathogenesis of arthritis remains unclear and there are no treatments that target mechanistic causes. Instead, treatments focus on symptom alleviation by suppressing joint pain and inflammation. Accordingly, pharmaceutical interventions include analgesic, steroidal anti-inflammatory drugs, and nonsteroidal anti-inflammatory drugs.

Complementary to traditional pharmaceutical treatments, nutritional interventions, that is, nutraceuticals, are an ongoing strategy for managing and preventing chronic disorders like OA. Nutraceuticals, such as glycosaminoglycans and certain botanical extracts, provided some improvement in pain and functional indices and a decrease in the loss of joint space width, whereas other studies did not.^3^ In addition, various collagen-based ingredients appear to be mildly effective in managing OA-associated symptoms,^4–7^ although replication of these results in placebo-controlled double-blind studies is needed to confirm beneficial effects. Bioactive compounds currently in use for the (supposed) alleviation of joint discomfort include collagen, glucosamine, and hyaluronic acid. Interestingly, these compounds occur naturally in high amounts in chicken eggshell membrane (EM), that is, the membrane located between the calcified shell and the egg white in chicken eggs. EM is primarily composed of fibrous proteins such as collagen type I that form the mesh-like structure of the bilayered material. Furthermore, EM contains the bioactive glycosaminoglycans dermatan sulfate, chondroitin sulfate, and hyaluronic acid. Due to its composition, EM may promote joint health and reduce pain and stiffness caused by OA by supplementing important anabolic factors lacking in today's typical Western diet and by engaging with inflammation through immunomodulation (oral tolerance).

Preclinical investigations with EM to date largely focused on immunomodulation properties with encouraging results reported so far.^8–11^ Also, a limited though growing body of evidence has surfaced over the last 5–10 years showing relief of OA complaints by EM.^12–17^ However, many of these human trials lack sufficient sample sizes and placebo-controlled double-blind randomized designs. This study is a critical test of the efficacy of a mildly processed form of EM for arthritis pain relief as it is the first double-blind randomized test comparing the longitudinal effects of EM intervention against a placebo in a large group of adult subjects diagnosed with knee OA.

## Materials and Methods

### Investigated products

Powdered chicken EM from a single production batch was kindly donated by the producer (DEPP B.V., Drachten, The Netherlands). During production of liquid egg products, membranes were separated from chicken egg shells by a mild water-based extraction process without any heat treatment though very effective in reducing any microbiological load and keeping the proteins in a native state. The EMs were carefully checked for food consumption through microbiological analysis. Membranes were subsequently dried, and ground into a fine powder (Eggbrane^®^). Doses of 300 mg powdered EM were then filled in white, opaque, gelatine capsules (size 00). Identical capsules were filled with 300 mg maltodextrin powder to obtain placebo capsules.

### Population

Subjects were recruited through various patient communities and health care organizations, by means of face-to-face notification, flyers, and advertisements in local news media. Inclusion criteria were age (40–75), a positive OA diagnosis of the knee, and a self-reported knee pain score of at least 3 and <9 on a 0–10 Numerical Rating Scale Pain (NRS-P; 0 = no pain at all, 10 the most intense pain imaginable). Furthermore, applicants were excluded in case they reported known allergy to eggs or egg products, were pregnant or breastfeeding, had previously enrolled in a study to evaluate pain relief <6 months before the study, or had participated in a study involving an investigational product (drug, device, or biologic) or a new application of an approved product. All subjects gave written informed consent before data acquisition.

Before enrolment subjects were instructed how and when to consume the product and how to use the online diary and questionnaires. Throughout the study, support through telephone and e-mail was available.

### Design

The study was designed as a randomized, double-blind, placebo-controlled nutritional intervention trial.

### Sample size, randomization, and treatment group composition

To allow for the detection of the clinically meaningful effect size of 1 NRS-P score point difference between the treatment group and the placebo group, minimal required sample sizes were calculated from knee OA NRS-P results reported by Verkleij *et al.*^18^ Applying their observed pooled standard deviation of 2.1, an effect size of 1.0 NRS-P score point requires a minimum of 63 subjects per treatment group, at a power of 80% and significance level of 5%. To compensate for a 15–20% loss to follow-up, it was decided to include 75 subjects per treatment group.

The random group assignments of the 150 subjects placed 40 male subjects (*M*_age_ = 63.8, standard deviation [SD] = 12.7) and 35 female subjects (*M*_age_ = 62.6, SD = 8.0) in the EM group, and 30 male subjects (*M*_age_ = 61.6, SD = 8.0) and 45 female subjects (*M*_age_ = 64.8, SD = 10.2) in the placebo group. Reported ages are based on subjects' ages on day 1 of the trial.

### Procedure

In the week before the start, subjects received written instructions and product capsules by postal mail. Starting on day 1 and continued over 12 weeks, subjects ingested one capsule per day containing EM or placebo. They could do this at a time and place of their choice. Once per week, and starting one day before ingesting the first capsule (day 0), subjects had to report NRS-P scores for the week passed (0–10, 0 = no pain at all, 10 = the most intense pain imaginable) through a short online questionnaire. In addition, subjects had to fill out an online version of the Knee injury and Osteoarthritis Outcome Scores (KOOS) questionnaire on days 0, 10, 21, 42, and 84. The KOOS questionnaire^19^ guides subjects in quantifying the perceived severity of knee OA-related complaints categorized in five subscales: joint pain during different activities (KOOS-Pain; 9 questions), the perceived severity of symptoms related to stiffness, swelling, grinding, locking, stretching, and bending of the knee (KOOS-Symptoms; 7 questions), the extent to which various daily life functioning is hindered by joint pain (KOOS-Daily Life; 17 questions), the extent to which recreational and sport functioning is hindered by joint pain (KOOS-Sport Rec; 5 questions), and the extent to which quality of life is affected by knee problems (KOOS-Quality; 4 questions). In addition, subjects were asked to keep daily online diaries with specified items on: product intake compliance, use of painkillers, adverse events, and occurrences deviating from normal daily routine such as unusual physical efforts, traveling *etc.* In case of (serious) adverse events, subjects were instructed to immediately contact their general practitioner, or the independent physician that was informed on the details of the intervention study.

This study was approved by the Independent Review Board Nijmegen, The Netherlands (#NL7309; clinical trial register number: NL64636.072.18).

### Outcomes/endpoints

The primary study outcomes consist of NRS-P scores assessed at weeks 0, 1, 2, 3, 4, 5, 6, and composite scores for the five KOOS categories “Pain”, “Symptoms”, “Daily Life”, “Sport Rec”, and “Quality” on days 0, 10, and 21. Secondary outcomes include NRS-P at weeks 7, 8, 9, 10, 11, 12, as well as composite KOOS scores in the five categories on days 42 and 84. Tolerance of daily consumption of EM was assessed as well through the daily diary.

### Illness (nausea) and dropouts

Throughout the study, subjects reported illnesses and other adverse events through online diaries. Most reports consisted of increasing knee pain and flu or common cold-related complaints. However, during December 2018 frequencies of reported nausea increased remarkably. From an initial 1–4 reports per month during September–November 2018, reports increased to 12 per month during December 2018 and January 2019. Consequently, nausea reports were closely monitored throughout the remainder of the study as these could, theoretically, be caused by the treatment. Reports returned to baseline in February 2019 and the research team decided not to intervene. In retrospect, the observed nausea reports coincided with high incidences of Respiratory Syncytial Virus (RSV) and Influenza-A infections in The Netherlands, as reported by the Dutch National Institute for Public Health and the Environment. These infections are probable causes of the nausea reports. Had nausea been caused by product spoilage, a steady increase of complaints would be expected rather than the observed bell-shape incidence curve in the midst of an Influenza-A and RSV outbreak in The Netherlands. Further support for this attribution comes from the fact that the placebo group and the EM group were equally represented in the nausea reports.

Because of the experienced nausea, two subjects decided to stop participating in the study (one from the placebo group and one from the EM group). Three subjects failed to start the study and 11 more dropped out at another point during the study. Of all dropouts, eight (three in the EM group, five in the placebo group) dropped out before producing all primary scores, and eight (six in the EM group and two in the placebo group) dropped out before producing all secondary scores.

### Statistical analyses

All analyses were performed according to the intention-to-treat principle, respecting the random treatment group assignments. Raw KOOS Likert-scale scores were coded 0 for the lowest category (mildest pain, symptoms, or hindrances), and incremented stepwise up to the score 4 for the highest category (most intense pain, symptom, or hindrances). KOOS subscale scores, consisting of *n*_s_ scores per subscale, were then calculated by


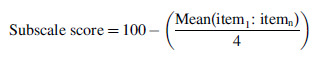


KOOS subscale scores are composite responses to specified questions within the categories “Pain”, “Symptoms”, “Daily Life”, “Sport Rec”, and “Quality”. Higher composite scores represent lower complaint intensities within these categories. Hence, a maximum score of 100 on the Symptoms composite score indicates a complete relief of Symptoms related to knee OA.

NRS-P scores and KOOS subscale scores were tested with repeated-measures analysis of variance for the main effects of Time (within-subject, for weeks 0–6 and weeks −12, respectively) and Treatment (between-subject, Placebo vs. EM) and the Time × Treatment interactions.

## Results

### NSR-P scores

**FIG. 1.**
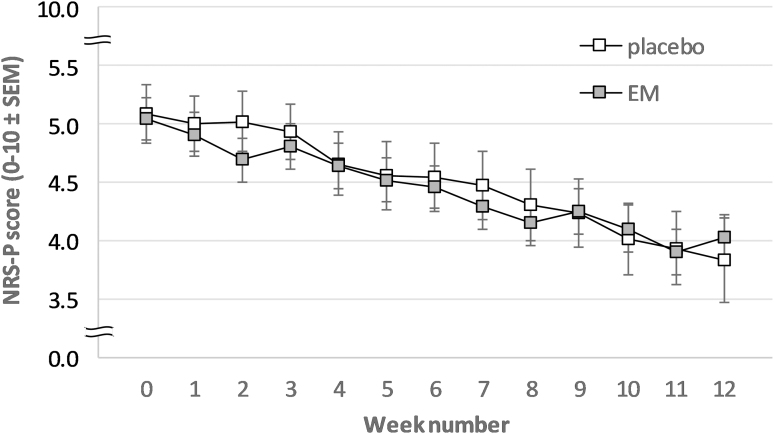
Weekly averaged (±standard error of the mean) NRS-P scores from EM and placebo group. Week 0 represents the baseline measurement 1 day before starting the 12-week intervention. EM, eggshell membrane; NRS-P, Numerical Rating Scale Pain.

At day 0, both groups produced equal average general pain (NRS-P) scores for that week of 5.0 (EM) and 5.1 (placebo), which is expected considering that OA patients were randomly assigned to treatment groups. During the first six treatment weeks (primary outcomes), NRS-P scores decreased for both groups at approximately the same rate (Fig. 1). Overall, this decrease was significant [*F*(6, 840) = 2.50; *P* < .05]. The lower NRS-P scores that were produced by the EM group during weeks 1, 2, and 3 did not produce a significant Time × Treatment interaction [*F*(6, 840) = 0.60; *P* = .73]. Secondary outcomes confirm these observations as the overall decline of NRS-P scores is consolidated [*F*(12, 1680) = 7.33; *P* < .001] and no Time × Treatment interaction is observed over 12 weeks either [*F*(12, 1680) = 0.48; *P* < .92].

#### Pain

**FIG. 2.**
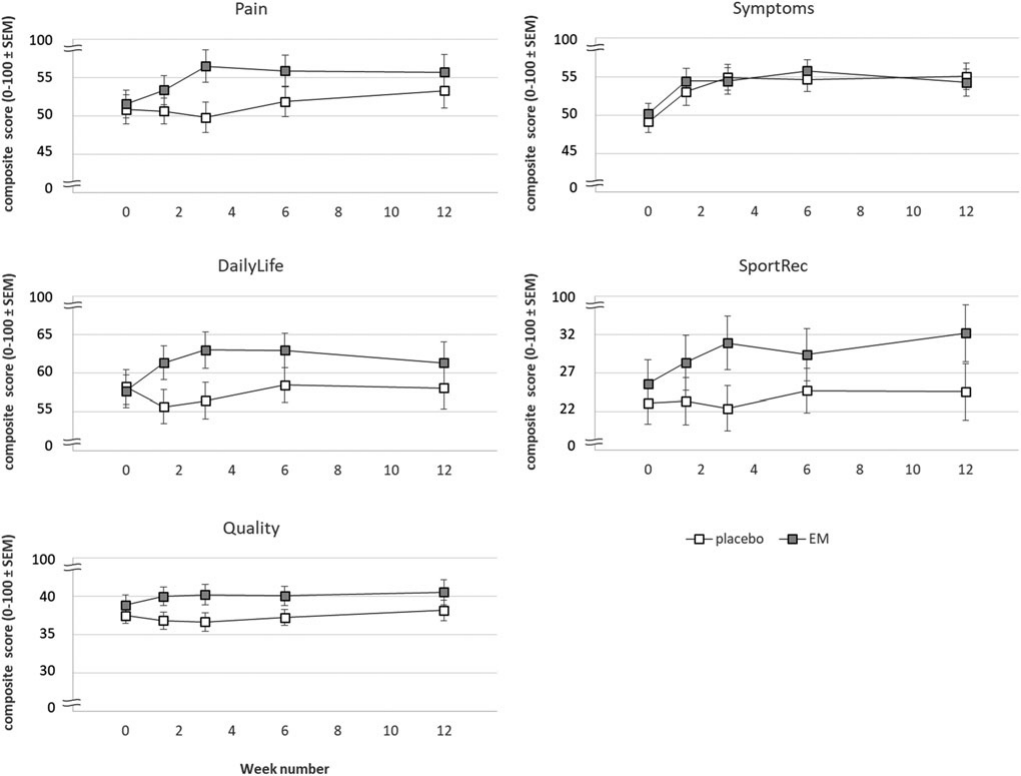
Average KOOS composite scores (±standard error of the mean) for the categories “Pain”, “Symptoms”, “DailyLife”, “SportRec,” and “Quality” compared between the placebo and the EM group. KOOS, Knee injury and Osteoarthritis Outcome Scores.

Primary KOOS “Pain” scores were nearly identical for both groups on day 0, but scores diverge over days 10 and 21 (Fig. 2). This divergence is mainly due to increasing scores (increasing relief of specific pain complaints) in the EM treatment group, while KOOS “Pain” scores decrease slightly (*i.e.*, a slight increase of combined specific pain complaints) in the placebo group. This divergence of scores between treatment groups is reflected in a significant Treatment × Time interaction [*F*(2, 264) = 7.13; *P* < .001]. No significant main effects of Time nor Treatment were observed. Secondary scores on days 42 (week 6) and 84 (week 12) showed a gradual convergence of KOOS “Pain” scores of both treatment groups. Overall, KOOS Pain (relief) scores appear higher for the EM treatment group (more pain relief), but this difference was not significant [*F*(1, 118) = 1.31; *P* = .25]. The Treatment × Time interaction is also significant for primary and secondary measures combined [*F*(4, 472) = 3.19; *P* < .05]. The main overall Time effect (overall change of scores over repeated measurements) was not significant [*F*(4, 472) = 2.21; *P* = .067].

#### Symptoms

In line with “Pain” scores, KOOS “Symptoms” composite scores were nearly identical for treatment groups on day 0. Interestingly, for both treatment groups, Symptoms scores increased over the remainder of the study (Fig. 2), without diverging. This observation is supported by a strong overall Time effect on primary scores [*F*(2, 264) = 20.9; *P* < .001] and primary and secondary scores combined [*F*(4, 472) = 13.5; *P* < .001]. No main effects of Time, nor Treatment were observed for primary scores and primary and secondary scores combined.

#### Daily life

Primary daily-life-functioning scores (KOOS: Daily Life) were identical for both groups on day 0 (Fig. 2) and diverged over days 10 and 21, mostly due to increased Daily Life scores in the EM group. This effect is supported by a strong Treatment × Time interaction [*F*(2, 264) = 9.39; *P* < .001]. Although Daily Life scores for both treatment groups converge slightly over secondary scores on days 42 (week 6) and 84 (week 12), the EM group consistently scores higher than the placebo group. Also for primary and secondary scores combined (over the full 12 weeks) the Treatment × Time interaction is significant [*F*(4, 472) = 3.66; *P* < .01]. No main effects of Time nor Treatment were observed for primary scores and for primary and secondary scores combined.

#### Sport Rec

KOOS scores in the Sport and Recreation category showed the same trends as Daily Life and Pain scores: divergence of primary scores in the EM group and the placebo group followed by a slight convergence over secondary scores (Fig. 2). However, these apparent trends did not result in significant test results. Only the initial divergence of primary scores, which resulted in Treatment × Time, nearly failed significance [*F*(2, 264) = 2.83; *P* = .060].

#### Quality

KOOS Quality-of-Life scores also showed similar trends as Pain, Daily Life, and Sport Rec scores, but failed to reach significance.

## Discussion

This double-blind placebo-controlled study demonstrated that Eggbrane, an EM extract obtained through a water-based process without any heat treatment, successfully relieves knee OA pain. At the applied dosage and compared against a placebo, EM relieved self-reported pain scores obtained with the KOOS questionnaire starting one week after initiation of treatment. This effect was significant for two of five KOOS category scores, that is, “Pain” and “Daily Life” functioning, aggregate pain, and functioning scores composed of complaint ratings for a wide variety of activities (*e.g.*, getting out of bed, walking, standing up from the toilet). These scores showed long-lasting improvement of 5–8 points on a 0–100 scale of complaint categories. The observed pain relief effects maximized after 3 weeks and decreased only slightly until measurements finished in week 12. Similar trends were observed for the Sport and Recreation functioning and the Quality-of-Life category scales, although not statistically significant.

In spite of the profound and lasting pain relief demonstrated by KOOS category scores, no EM-induced pain relief was observed for the generic numerical pain rating scale (NRS-P). Instead, we observed a consistent decrease of NRS-P scores over time for both treatment groups: patients benefited equally well from the placebo and the EM, suggesting a placebo effect. The 11-point NRS-P is a commonly used instrument to quantify the pain experienced by patients in a wide variety of settings. For instance, NRS-P measures proved consistent and reliable for the assessment of rheumatoid pain, and results correlated well with those obtained with alternative scales.^20^ In this light, the consistent and significant improvement of two-out-of-five KOOS category scores due to EM treatment (and similar trends for two more KOOS category scores) appeared incompatible with the NRS-P observations. KOOS categories that showed significant treatment effects (*i.e.*, KOOS-Pain and KOOS-Daily Life categories) gauged recalled pain and impaired functioning during specific activities like getting out of bed, standing up from the toilet, walking the stairs, or performing an exercise. Instead, KOOS rating scales that do not refer to activities, but focus on symptoms only (*i.e.*, the KOOS-Symptoms category) and NRS-P results produce a placebo effect. Although NRS-P was validated in studies employing the immediate quantification of acute pain,^21^ NRS-P was not sensitive for treatment effects in the present study. Apparently, to reveal treatment effects on OA knee pain by retrospect self-reports, questions need to address the conditions under which pain may have been perceived.

EM contains various potential anabolic factors lacking in today's typical Western diet, and as well engage with inflammation through immunomodulation as described in the section “Introduction”. Eggbrane is carefully extracted and mildly processed to provide a high amount of potential bioactives, especially proteinaceous components, in its most native state. Further in-depth study is required to link the observed benefits to its specific composition.

Half-way the intervention study, a notable yet not alarming raise was observed in the frequency at which participants mentioned nausea in their daily reports. These incidences kept pace with a nation-wide influenza-A and RSV epidemic, which was a plausible external source for the nausea symptoms. Had nausea been caused by the composition of one of the two capsules (EM or placebo), a systematic bias of study outcomes would have been possible because of the occurrence of additional symptoms in that treatment group, or because of dropouts in that treatment group. In retrospect, this possibility is ruled out because of the equal division of nausea reports between treatment groups. For both the primary scores and the secondary scores, the number of remaining subjects are well above the acceptable minimum of 63 subjects per treatment condition.

About half a dozen earlier clinical studies have been reported using a certain form of EM. Most of these studies have been either open label studies and/or of a preliminary character, largely downgrading the value of the outcomes.^12,14–17^ Ruff *et al.*^13^ reported a double-blind placebo-controlled trial that did show an absolute rate of response of EM in OA that was statistically significant (up to 26.6%) versus placebo at all time points for both pain and stiffness, but no significantly improved function and overall Western Ontario and McMaster Universities scores, although trending toward improvement. Rapid responses were seen for mean pain subscores (15.9% reduction, *P* = .036) and mean stiffness subscores (12.8% reduction, *P* = .024) occurring after only 10 days of supplementation. Notably, interest for EM as OA treatment has grown as shown by more recent studies. A double-blind controlled study showed rapid improved recovery from exercise-induced joint pain (8 days) and stiffness (4 days) and reduced discomfort immediately following exercise.^22^ In another double-blind, placebo-controlled clinical trial involving 88 adults with OA,^23^ the ingestion of an EM hydrolysate significantly enhanced average individual physical capacity (walking distance and ability) and reduced stiffness by the fifth day of supplementation. These effects were maintained over 12 weeks. The results of the present study provide consistent support of the potential of EM to provide fast and sustainable relief in subjects dealing with OA.

World-wide, thousands of tons of eggshells are produced annually as a byproduct of the poultry industry. Disposal of these eggshells creates an environmental and financial burden and, therefore, alternative uses for these materials would be of obvious benefit. The demonstrated efficacy of mildly processed EM (Eggbrane) to alleviate pain in a population suffering from OA of the knee offers a combination of affordable health care and sustainable use of food industry waste.

## References

[1] Johnson VL, Hunter DJ: The epidemiology of osteoarthritis. Best Pract Res Clin Rheumatol 2014;28:5–15 24792942 10.1016/j.berh.2014.01.004

[2] Vos T, Flaxman AD, Naghavi M, *et al.*: Years lived with disability (YLDs) for 1160 sequelae of 289 diseases and injuries 1990–2010: A systematic analysis for the global burden of disease study 2010. Lancet 2012;380:2163–2196 23245607 10.1016/S0140-6736(12)61729-2 PMC6350784

[3] Ragle RL, Sawitzke, AD: Nutraceuticals in the management of osteoarthritis: A critical review. Drugs Aging 2012;29:717–731 23018608 10.1007/s40266-012-0006-3

[4] Bello AE, Oesser S: Collagen hydrolysate for the treatment of osteoarthritis and other joint disorders: A review of the literature. Curr Med Res Opin 2001;22:2221–2232 10.1185/030079906X148373 17076983

[5] Schauss AG, Stenehjem J, Park J, Endres JR, Clewell A: Effect of the novel low molecular weight hydrolyzed chicken sternal cartilage extract, BioCell Collagen, on improving osteoarthritis-related symptoms: A randomized, double-blind, placebo-controlled trial. J Agric Food Chem 2012;60:4096–4101 22486722 10.1021/jf205295u

[6] Kumar S, Sugihara F, Suzuki K, Inoue N, Venkateswarathirukumara S: A double blind, placebo controlled, randomised, clinical study on the effectiveness of collagen peptide on osteoarthritis. J Sci Food Agric 2015;95:702–707 24852756 10.1002/jsfa.6752

[7] Van Vijven JP, Luijsterburg PA, Verhagen AP, van Osch GJ, Kloppenburg M, Bierma-Zeinstra SM: Symptomatic and chondroprotective treatment with collagen derivatives in osteoarthritis: A systematic review. Osteoarthritis Cartilage 2012;20:809–821 22521757 10.1016/j.joca.2012.04.008

[8] Benson KF, Ruff KJ, Jensen GS: Effects of natural eggshell membrane (NEM) on cytokine production in cultures of peripheral blood mononuclear cells: Increased suppression of tumor necrosis factor-alpha levels after in vitro digestion. J Med Food 2012;15:360–368 22168811 10.1089/jmf.2011.0197 PMC3308710

[9] Ruff KJ, DeVore DP: Reduction of pro-inflammatory cytokines in rats following 7-day oral supplementation with a proprietary eggshell membrane-derived product. Mod Res Inflamm 2014;3:19–25

[10] Ruff KJ, Durham PL, O'Reilly A, Long FD: Eggshell membrane hydrolyzates activate NF-kB in vitro: Possible implications for in vivo efficacy. J Inflamm Res 2015;8:49–57 25709492 10.2147/JIR.S78118 PMC4332312

[11] Wedekind KJ, Ruff KJ, Atwell CA, Evans JL, Bendele AM: Beneficial effects of natural eggshell membrane (NEM) on multiple indices of arthritis in collagen-induced arthritic rats. Mod Rheumatol 2017;27:838–848 27846748 10.1080/14397595.2016.1259729

[12] Ruff KJ, DeVore DP, Leu MD, Robinson MA: Eggshell membrane: A possible new natural therapeutic for joint and connective tissue disorders. Results from two open-label human clinical studies. Clin Interv Aging 2009;4:235–240 19554094 10.2147/cia.s5797 PMC2697588

[13] Ruff KJ, Winkler A, Jackson RW, DeVore DP, Ritz BW: Eggshell membrane in the treatment of pain and stiffness from osteoarthritis of the knee: A randomized, multicenter, double-blind, placebo-controlled clinical study. Clin Rheumatol 2009;28:907–914 19340512 10.1007/s10067-009-1173-4 PMC2711914

[14] Danesch U, Seybold M, Rittinghausen R, Treibel W, Bitterlich N: NEM^®^ brand eggshell membrane effective in the treatment of pain associated with knee and hip osteoarthritis: Results from a six center, open label German clinical study. J Arthritis 2014;3:136

[15] Brunello E, Masini A: NEM^®^ brand eggshell membrane effective in the treatment of pain and stiffness associated with osteoarthritis of the knee in an Italian study population. Int J Clin Med 2016;7:169–175

[16] Aguirre A, Gil Quintana E, Fenaux M, Erdozain S, La Nuez M: Effects of 50 days Ovomet^®^ supplementation on biochemical parameters and subjective pain perception among old institutionalized patients. A preliminary study. J Osteoporos Phys Act 2017;5:198

[17] Aguirre A, Gil Quintana E, Fenaux M, Erdozain S, La Nuez M: Effects of 50 days eggshell membrane Ovomet^®^ supplementation on biochemics parameters and subjective pain perception among crossfit athletes. A preliminary study. J Trauma Treat 2017;6:371

[18] Verkleij SP, Luijsterburg PA, Willemsen SP, Koes BW, Bohnen AM, Bierma-Zeinstra SM: Effectiveness of diclofenac versus paracetamol in knee osteoarthritis: A randomised controlled trial in primary care. Br J Gen Pract 2015;65:e530–e537 26212849 10.3399/bjgp15X686101 PMC4513741

[19] Roos, EM, Roos HP, Lohmander LS, Ekdahl C, Beynnon BD: Knee Injury and Osteoarthritis Outcome Score (KOOS)-development of a self-administered outcome measure. J Orthop Sports Phys Ther 1998;28:88–96 9699158 10.2519/jospt.1998.28.2.88

[20] Downie WW, Leatham PA, Rhind VM, Wright V, Branco JA, Anderson, JA: Studies with pain rating scales. Ann Rheum Dis 1978;37:378–381 686873 10.1136/ard.37.4.378 PMC1000250

[21] Bijur PE, Latimer CT, Gallagher EJ: Validation of a verbally administered numerical rating scale of acute pain for use in the emergency department. Acad Emerg Med 2003;10:390–392 12670856 10.1111/j.1553-2712.2003.tb01355.x

[22] Ruff KJ, Morrison D, Duncan SA, Back M, Aydogan C, Theodosakis J: Beneficial effects of natural eggshell membrane versus placebo in exercise-induced joint pain, stiffness, and cartilage turnover in healthy, postmenopausal women. Clin Interv Aging 2018;13:285–295 29497287 10.2147/CIA.S153782 PMC5822842

[23] Hewlings S, Kalman D, Schneider LV: A randomized, double-blind, placebo-controlled, prospective clinical trial evaluating water-soluble chicken eggshell membrane for improvement in joint health in adults with knee osteoarthritis. J Med Food 2019;22:875–884 31381494 10.1089/jmf.2019.0068 PMC6748399

